# The effect of reducing dietary energy density via the addition of water to a dry diet, on body weight, energy intake and physical activity in adult neutered cats*

**DOI:** 10.1017/jns.2014.22

**Published:** 2014-09-25

**Authors:** Janet E. Alexander, Alison Colyer, Penelope J. Morris

**Affiliations:** WALTHAM^TM^ Centre for Pet Nutrition, Waltham-on-the-Wolds, Melton Mowbray LE14 4RT, UK

**Keywords:** Energy density, Cats, Body weight, Food intake, Physical activity, IMER, individual maintenance energy requirements, sbw, starting body weight, tmc, total moisture content

## Abstract

Increasingly domestic cats live in an overfeeding and underexercising environment where obesity is a major health concern. One strategy to aid healthy body weight maintenance is dietary energy dilution. Published data indicate that increasing dietary moisture content leads to a reduction in energy intake and increased activity. However, a number of different methodologies were employed in these studies and associated changes in physical activity have only been measured once. The aim of the present study was to determine the effect of diets of three different moisture contents offered in excess of energy requirements, on body weight, energy intake and physical activity in adult neutered cats. Sixty-nine adult cats randomised into three groups, received 100 % of their daily individual maintenance energy requirements (IMER) of dry diet or dry diet hydrated to 40 or 80 % total moisture content (tmc). Baseline activity, intake, body weight and body composition were measured. Following this baseline phase, the cats received the same diets at 200 % of daily IMER and the measurements repeated over the next 28 d. When offered the diets at 200 % IMER, cats fed the dry diet significantly increased body weight and percentage of body fat (*P* < 0·01), while those offered the hydrated diets did not (*P* > 0·01). The levels of physical activity in cats offered the hydrated 80 % tmc diet were significantly (*P* < 0·01) higher than those offered the dry or 40 % tmc diet. We suggest that dietary energy dilution by addition of water may be a useful strategy for healthy body weight maintenance in overfed cats.

Domestic cats often live in an overfeeding environment and as such, it has been estimated that approximately 35 % of the adult cat population is overweight^(^^1^^,^^2^^)^. Strategies are therefore required to enable cats to maintain a healthy body weight even when excess food is available. One possible strategy is the reduction of dietary energy density by dilution with non-nutritive substances. Published data indicate that cats do not increase their energy intake to fully compensate for energy dilution of the diet and therefore gain less body weight than those offered the undiluted diet in excess of maintenance energy requirements^(^^3^^–^^6^^)^. Two of these studies have compared the effects of energy dilution by the addition of water to diets of a fixed nutritional composition^(^^5^^,^^6^^)^. Both energy intake and body weight were significantly lower in cats fed a canned diet containing 80 % moisture compared with when fed *ad libitum* the same diet freeze dried to a moisture content of 10 %, activity levels were not determined^(^^6^^)^. Furthermore, Cameron *et al.*^(^^5^^)^ reported that when water was added to a dry diet to achieve a total moisture content (tmc) of 50 %, adult cats gained less total body mass compared with cats offered the same number of joules of the same dry diet (10 % tmc) with no additional hydration. These authors suggested that the difference could be attributed to reduced energy intake as well as increased energy expenditure in the form of physical activity in the cats fed the hydrated diet. However, previous weight gain and loss in this cohort may have influenced their levels of physical activity^(^^5^^)^.

Collectively these data suggest that alterations in dietary energy density via manipulation of moisture content could be a useful strategy for weight management in overfed cats. However, the effect of a range of dietary moisture contents on body weight, composition, energy intake in conjunction with physical activity has not previously been assessed. The aim here was to determine the effect of three levels of dietary moisture on these parameters when food amounts in excess of energy requirements were offered to adult neutered cats which had never been overweight.

## Experimental methods

The study was of a randomised parallel design with twenty-three cats (nine females and fourteen males) aged between 14 and 21 months, assigned to each treatment group. Cats were examined by a veterinarian at the start of the study and deemed to be within 5 % of ideal body weight and clinically healthy. The cats were group housed according to the treatment in social rooms with free access to water except when, twice daily for a period of 30 min, they were individually housed for feeding. The 20 min human socialisation sessions were fixed prior to the start of the study. This work was approved by the WALTHAM Ethics Committee and followed United Kingdom Home Office Code of Practice guidelines for animal welfare.

Test diets were produced by addition of water to a commercial dry diet (Royal Canin Fit 32) from a single batch conforming to National Research Council Nutrient Guidelines 2006^(^^7^^)^: as is 59 g/kg moisture, 326 g/kg protein, 144 g/kg fat, 55 g/kg crude fibre, 71 g/kg ash, calculated^(^^7^^)^ metabolisable energy 15 755 kJ/kg. The required amount of kibble and water were soaked in a sealed tub at room temperature overnight to achieve a tmc of: 6; 40; or 80 %. Evaporation from the diets between preparation and the end of feeding time was <2 % of the total mass (unpublished results).

During phase 1 (weeks 1–4) cats were offered 100 % of their individual maintenance energy requirements (IMER) daily, in two meals. In phase 2 (weeks 5–8), cats were offered the same diets at 200 % of their IMER daily, in two meals. IMER was determined as the number of kJ/d observed to maintain each individual within 5 % of their ideal body weight and body condition score^(^^8^^)^ for 8 weeks prior to the start of the study.

### Measures

Body weight was recorded in kg, weekly in the fasted (>12 h) state on a calibrated scale (±0·1 g, Sartorius Signum 2 top-pan balance).

Individual intake was recorded on a calibrated scale (±0·01 g, Sartorius IB31000P top-pan balance), as mass (g) of diet offered minus mass (g) of diet refused per d.

Physical activity was recorded using Actical™ accelerometers (Philips Healthcare) attached to each individual cat's collar. Cats were habituated to wearing these prior to the start of the study. Data were recorded for 72 h periods in week 3 of both phases. Actical™ accelerometers have been previously validated for use in cats^(^^9^^)^.

Body composition was assessed in fasted cats in week 2 of phase 1 and week 4 of phase 2 by means of dual-energy X-ray absorptiometry (Lunar Prodigy; GE Healthcare). Cats were sedated according to the protocol described in Cameron *et al.*^(^^5^^)^ and dual-energy X-ray absorptiometry has been previously validated in cats^(^^10^^)^.

### Statistical analysis

Final body weight was the primary outcome and it was determined that twenty cats per group were required for the study to detect a difference of 3 % between groups with 90 % power and a 5 % significance level. Linear mixed model analysis allowing for repeat measures was carried out using the nlme and multcomp packages of R v2.15.0 statistical software^(^^11^^)^ as detailed later. Bonferroni adjustments were made for the inclusion of four endpoints and therefore the significance value used was *P* < 0·05/4 = *P* < 0·0125.

### Body weight

Cat was fitted as the random effects model and residuals fitted with an autoregressive correlation structure to order 1. Initial body weight was used as a baseline covariate. Time, diet and their interaction were fitted as fixed effects.

### Activity

Activity data were log_10_ transformed prior to analyses. Phase, nested in cat, was fitted as the random effects model. Phase, diet and their interaction were fitted as the fixed effects.

### Intake

Week, nested in cat, was fitted as the random effects model and diet, week and their interaction as the fixed effects.

### Body fat

Cat was fitted as the random effect but no correlation structure was used. Phase, diet and their interaction were fitted as the fixed effects.

## Results

Prior to the start of the study one cat was removed for unrelated health reasons; therefore the 6 % tmc group comprised twenty-two (nine females and thirteen males) rather than twenty-three cats.

### Body weight and composition

**Fig. 1.**
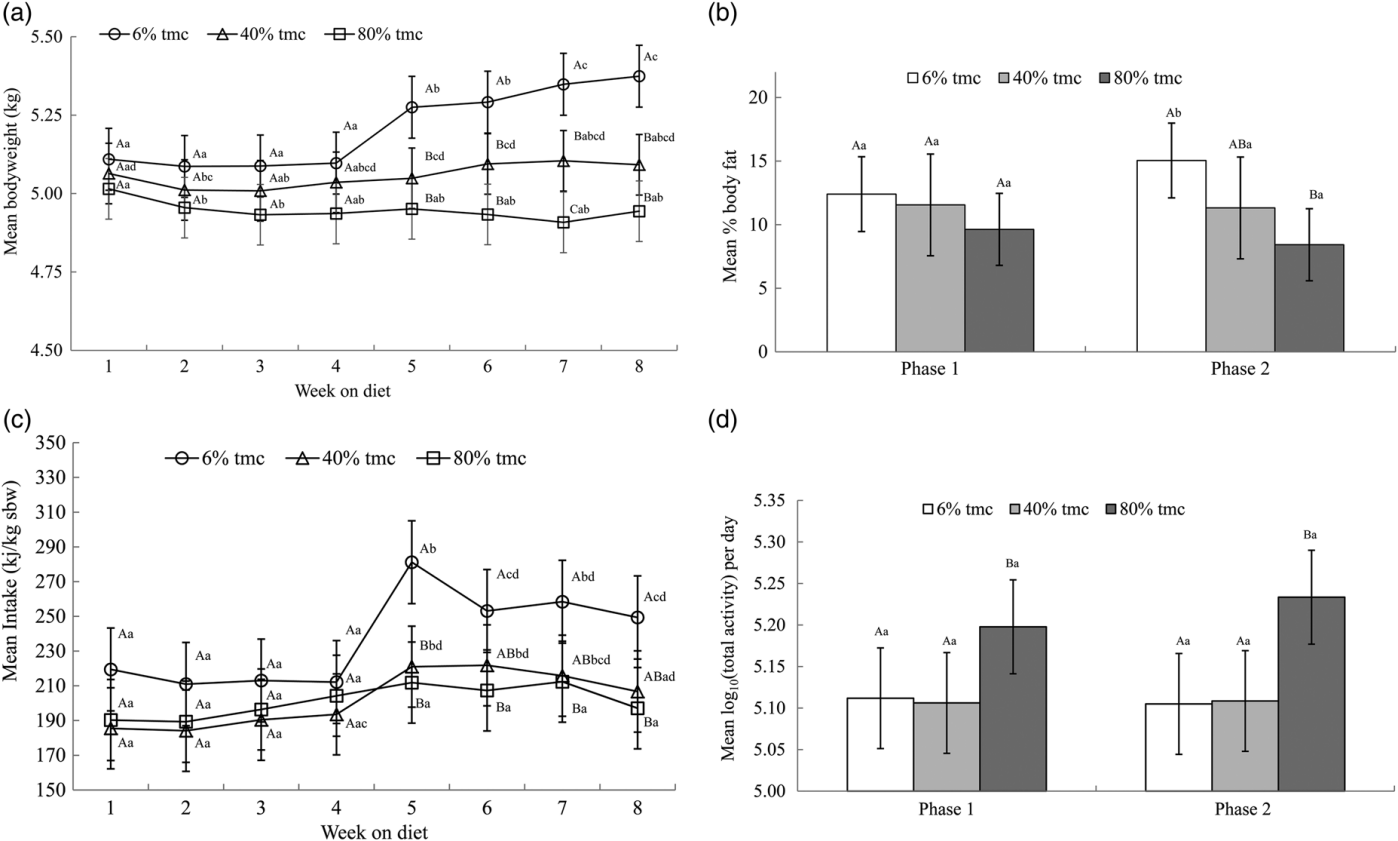
(a) Body weight. Mean bodyweight (kg), adjusted for starting bodyweight at day 1, with 95 % familywise CI; *n* 22–23. Total moisture content (tmc). A, B, C, between diets, a, b, c within diets, mean values with unlike letters were significantly different (*P* < 0·01). (b) Body fat. Mean % body fat, with 95 % familywise CI; *n* 22–23. Total moisture content (tmc). A, B, C, between diets, a, b, c within diets, mean values with unlike letters were significantly different (*P* < 0·01). (c) Intake. Mean intake (kJ), adjusted for starting body weight (kg sbw) at day 1, with 95 % familywise CI; *n* 22–23. Total moisture content (tmc). A, B, C, between diets, a, b, c within diets, mean values with unlike letters were significantly different (*P* < 0·01). (d) Physical activity. Log_10_ mean total activity (U/d), with 95 % familywise CI; *n* 22–23. Total moisture content (tmc). A, B, C, between diets, a, b, c within diets, mean values with unlike letters were significantly different (*P* < 0·01).

There was no significant (*P* > 0·01) difference in either body weight or percentage body fat between the diet groups during phase 1 of the study when fed 100 % of IMER (Fig. 1(a) and (b)). During phase 2, when offered 200 % IMER of the test diets, cats in the 6 % tmc group (*P* < 0·01) gained significant body weight (Fig. 1(a)) and body fat (Fig. 1(b)). Neither body weight nor body fat increased significantly (*P* > 0·01) when the 40 % tmc and 80 % tmc diets were offered in excess (Fig. 1(a) and (b)). No significant difference (*P* > 0·01) in body weight was seen between cats fed the 40 or 80 % tmc diet except in week 7 (Fig. 1(a)).

### Intake

Mean intake in terms of kJ/kg of starting body weight (sbw; kj/kg) was not significantly different between weeks within diet groups (*P* > 0·01) or between diet groups (*P* > 0·01) during phase 1 (Fig. 1(c)). In phase 2, when 200 % of IMER was offered, the mean intake of cats in the 6 % tmc group increased significantly (*P* < 0·01) from 212·17 kJ/kg sbw (95 % CI 188·32, 236·02) to 281·21 kJ/kg sbw (95 % CI 257·36, 305·06) and remained significantly higher for the duration of the phase (Fig. 1(c)). In week 5, the mean intake of the 40 % tmc group also increased significantly (*P* < 0·01) from 193·55 kJ/kg sbw (95 % CI 170·24, 216·90) to 221·00 kJ/kg sbw (95 % CI 197·69, 244·35). However, by week 8 the intake of this group had reduced to 206·73 kJ/kg sbw (95 % CI 183·30, 230·12) and was no longer significantly different from phase 1 (Fig. 1(c)). The mean intake of the 80 % tmc group did not change significantly at any time (Fig. 1(c)).

### Activity

There was no significant difference in the activity level of cats within diet group between phases (*P* > 0·01). However, the mean activity level of cats in the 80 % tmc group was significantly greater (*P* < 0·001) than that of the 6 and 40 % tmc groups in both phases by 28 % (95 % CI 9, 50) (Fig. 1(d)).

## Discussion

Several authors^(^^3^^–^^5^^,^^12^^)^ have demonstrated that cats do not increase their energy intake to fully compensate for energy dilution by the addition of water. Although a number of different levels of dietary moisture content have been reported to have an effect on energy intake in cats, only one (50 % tmc) has been associated with increased physical activity when compared with a dry (10 % tmc) diet^(^^5^^)^. However, these data may have been confounded by the cat's previous history of weight gain and loss. The aim of the present study was to elucidate whether diets of other dietary moisture contents could have an effect on body weight, energy intake and physical activity when cats of previously stable body weight were offered diets of three moisture contents in excess of IMER.

Cats offered the dry diet gained significantly more body weight than those fed the hydrated diets in excess of IMER. Increased body weight was accompanied by a significant increase in percentage of body fat as measured by dual-energy X-ray absorptiometry. These differences in body weight and composition are consistent with the published data^(^^5^^,^^6^^)^. Little difference in body weight was seen between cats fed a 40 % tmc diet and those fed an 80 % tmc diet (Fig. 1(a)). This may indicate that in overfed cats there is little benefit to healthy weight maintenance in increasing the moisture content of the diet above 40 %. However, it is possible that if this phase had been over a longer period or if the food were offered *ad libitum*, the difference between these groups may have become significant.

Differences in weight gain reflected differences in energy intake. When offered 200 % of IMER, cats offered the 80 % tmc diet had lower energy intake (kJ/kg sbw) than those offered the 6 % tmc diet. It is possible that some of the difference in intake could be attributed to diet palatability. However, intake (kJ/kg sbw) was not significantly different between diet groups during phase 1 when cats were offered 100 % IMER and cats offered the 80 % tmc diet ate significantly more grams of diet on a wet matter basis than those offered the 6 % tmc dry diet (data not shown). In phase 2, cats fed the 6 % tmc diet significantly increased their intake (kJ/kg sbw) when excess was offered, whereas those offered the 80 % moisture diet did not. This may suggest that the volume of diet a cat can eat in a single meal is a limiting factor and that those offered the 80 % tmc diet could not further increase the volume consumed. In cats, volume limitation has been demonstrated when the energy content of diets was reduced by the addition of the bulking agents cellulose and kaolin^(^^12^^,^^13^^)^. Dietary volume control is regulated by receptors in the stomach where vagal and splanchnic nerves detect distention sending signals to the brain that can terminate feeding^(^^14^^)^. This mechanism may restrict energy intake when a high tmc, low-energy density diet is consumed compared with a low tmc, high-energy density diet. In many species, such volume-led responses reduce energy intake over a limited period of time but this may not be the case in the cat. Skultety^(^^15^^)^ reported that compensation for reduced dietary energy density in cats takes 75–200 d to initiate. No increase in energy intake was seen in cats offered the hydrated diets over the 8 weeks of the present study. As a carnivore, the cat's natural diet is made up of prey which has a relatively constant energy density^(^^16^^)^. It is therefore possible to speculate that in the wild, cats do not require mechanisms to rapidly compensate for changes in dietary energy density. It has been reported in other species that when the energy density of the diet is decreased and the macronutrient profile remains constant, meal frequency may increase to ensure that energy intake remains constant^(^^17^^)^. However, such compensation for dietary energy dilution could not have been seen in the present study as a meal-feeding protocol was employed rather than feeding *ad libitum*.

Physical activity levels were significantly higher in cats fed the 80 % tmc diet when both 100 and 200 % of IMER was offered. However, as no activity recordings were taken prior to feeding the test diets it was therefore not possible to verify that cats fed the 80 % tmc diet had the same basal activity levels as the other two groups. The increased physical activity levels detected support previous findings^(^^5^^)^, which reported an increase in activity when cats were fed a 50 % tmc diet. In the present study, no effect was seen when a 40 % tmc diet was fed. This suggests that the ‘threshold’ for the effect of dietary moisture content on physical activity is greater than 40 % tmc but equal or below 50 % tmc. Both physiological and behavioural responses to dietary energy dilution could explain this, for example differences in the cat's relative level of hydration. It has been shown that cats fed a diet of higher water content have greater total fluid intake than those fed a lower water content diet, even when drinking water is available *ab libitum*^(^^18^^)^. In human subjects, mild dehydration has been shown to reduce physical performance and increase fatigue^(^^19^^)^ and these effects could manifest themselves as reduced levels of physical activity. Other physiological changes in response to dietary composition have been shown to affect physical activity levels. For example, several gut-derived peptides have been demonstrated to influence physical activity^(^^20^^,^^21^^)^. Peripheral administration of leptin to mice and human subjects resulted in increased physical activity, whereas administration of ghrelin to rats decreased activity levels^(^^20^^,^^21^^)^. Differential responses of these hormones to diets of varying moisture contents could be responsible for the activity changes seen.

At a time when the domestic cat population is suffering increasing levels of obesity, these data indicate the possibility of designing diets that aid healthy body weight maintenance by reducing intake and increasing activity. The findings of the present study offer a strategy for healthy body weight maintenance when cats are offered diet in excess.
